# Increased protein intake affects pro-opiomelanocortin (POMC) processing, immune function and IGF signaling in peripheral blood mononuclear cells of home-dwelling old subjects using a genome-wide gene expression approach

**DOI:** 10.1186/s12263-019-0654-6

**Published:** 2019-11-28

**Authors:** Gyrd O. Gjevestad, Kirsten B. Holven, Amanda Rundblad, Arnar Flatberg, Mari Myhrstad, Karina Karlsen, Shivaprakash J. Mutt, Karl-Heinz Herzig, Inger Ottestad, Stine M. Ulven

**Affiliations:** 1 Department of Nutrition, Institute of Basic Medical Sciences, University of Oslo, P.O. Box 1046, Blindern, 0317 Oslo, Norway; 2 Innovation and marketing, TINE SA, Lakkegata 23, 0187 Oslo, Norway; 3 National Advisory Unit on Familial Hypercholesterolemia, Department of Endocrinology, Morbid Obesity and Preventive Medicine, Oslo University Hospital, P.O. Box 4950 Nydalen, 0424 Oslo, Norway; 4 Department of Clinical and Molecular Medicine, Faculty of Medicine, Genomics Core Facility, Norwegian University of Sciences and Technology, Olav Kyrres gt. 9, 7489 Trondheim, Norway; 5 Faculty of Health Sciences, Department of Nursing and Health Promotion, OsloMet – Oslo Metropolitan University, P.O. Box 4 St. Olavs plass, 0130 Oslo, Norway; 6 Research Unit of Biomedicine, and Biocenter of Oulu, Oulu University Hospital and Medical Research Center Oulu, Oulu University, P.O Box 5000, 90014 Oulu, Finland; 7 Department of Gastroenterology and Metabolism, Poznan University of Medical Sciences, 60-572 Poznan, Poland

**Keywords:** Older adults, Randomized Controlled trial, Dairy protein, Peripheral mononuclear cells, Transcriptomics, Immune function, IGF-1, POMC

## Abstract

**Background:**

Adequate protein intake among older adults is associated with better health outcomes such as immune function and metabolic regulation of skeletal muscle, but conflicting results make it difficult to define the optimal intake. To further understand the impact of protein intake on metabolic processes, the aim of the study was to explore genome-wide gene expression changes in peripheral blood mononuclear cells (PBMCs) in home-dwelling old subjects after increased protein intake for 12 weeks.

**Method:**

In a parallel double-blind randomized controlled intervention study, subjects (≥ 70 years) received a protein-enriched milk (2 × 20 g protein/day, *n* = 14, mean (±SD) age 76.9 ± 4.9 years) or an isocaloric carbohydrate drink (*n* = 17, mean (±SD) age 77.7 ± 4.8 years) for breakfast and evening meal for 12 weeks. PBMCs were isolated before and after the intervention. Microarray analysis was performed using Illumina technology. Serum levels of gut peptides and insulin growth factor (IGF)-1 were also measured.

**Results:**

In total 758 gene transcripts were regulated after increased protein intake, and 649 gene transcripts were regulated after intake of carbohydrates (*p* < 0.05). Forty-two of these genes were overlapping. After adjusting for multiple testing, 27 of the 758 gene transcripts were regulated (FDR, *q*-value < 0.25) after protein intake. Of these 25 were upregulated and two downregulated. In particular, genes and signaling pathways involved in pro-opiomelanocortin (POMC) processing, immune function, and IGF signaling were significantly altered.

**Conclusions:**

PBMCs can be used to study gene expression changes after long-term protein intake, as many signaling pathways were regulated after increased protein intake. The functional significance of these findings needs to be further investigated.

**Trial registration:**

ClinicalTrials.gov, ID no. NCT02218333. The study was registered on August 18, 2014.

## Background

Adequate intake of dietary protein is important to maintain muscle mass and muscle strength in older adults. Age-related loss of skeletal muscle mass, skeletal muscle strength and functional performance is linked to a number of poor health outcomes, such as impaired functionality, reduced ability to manage activities of daily life [1, 2], reduced quality of life [3], increased morbidity, but also mortality [4–6]. Epidemiological studies have indicated that dietary protein intake may be a modifiable risk factor for loss of skeletal muscle mass and skeletal muscle strength (sarcopenia) in older adults [7]. However, no consensus has been reached related to recommendations of optimal intake level of protein in older adults, and results from studies investigating changes in muscle mass or muscle strength after increased protein intake have been conflicting [6, 8–11].

Dietary interventions usually cause only small changes within the body and may be difficult to detect by changes in the phenotype, such as muscle mass, muscle strength or circulating inflammatory markers, especially in short-term trials [12]. Other, more sensitive methods, such as changes in gene transcripts, may detect important changes at a molecular level. These signatures may serve as biomarkers in a life-long perspective, affecting homeostatic control and the risk of developing life style related diseases [12, 13]. Protein intake and certain amino acids are known to regulate gene expression [14, 15]. The most studied pathway regulated by dietary proteins is probably the mammalian target of rapamycin (mTOR) pathway, which is involved in the regulation of muscle protein synthesis [16]. However, proteins and amino acids are also shown to play an important role in the regulation of general control nonderepressible 2 (GCN2)/activating transcription factor 4 (ATF4), which regulate protein metabolism, but may also affect lipid and glucose metabolism [14].

Peripheral blood mononuclear cells (PBMCs) can be used as a model system for studying changes in gene expression levels in dietary intervention studies [17, 18]. PBMCs include monocytes, lymphocytes and natural killer (NK) cells mainly; cells that play an important role in inflammation and in the development of chronically related diseases, such as cardiovascular diseases [19, 20]. PBMCs are circulating cells exposed to nutrients, metabolites and peripheral tissues, and PBMCs may therefore reflect whole-body health [18]. However, their role in aging and age-related conditions, such as loss of muscle mass and the decline of immune function with aging is not well described. Furthermore, how gene expression in PBMCs is affected by dietary protein intake has hardly been investigated in older subjects. We recently showed that consumption of protein-enriched milk had minor effects on a limited number of selected inflammatory genes and genes involved in muscle mass in PBMCs from older adults, when using a targeted approach [21]. In the present study we further elucidated the impact of protein intake on metabolic processes. The aim of our study was to explore genome-wide gene expression changes in PBMCs in home-dwelling old subjects with reduced physical strength and/or performance after 12 weeks with increased protein intake.

## Results

### Subject characteristics

There were no statistically significant differences between the study groups in either of the parameters measured at baseline, such as BMI, lean body mass, hs-CRP or the amounts of monocytes and lymphocytes (Table 1).

**Table 1:** Baseline characteristics

	Protein group (*n* = 14)	Carbohydrate group (*n* = 17)
Gender (men/women)	4/10	5/12
Age (years)	76.9 ± 4.9	77.7 ± 4.8
BMI (kg/m^2^)	27.1 ± 3.8	26.4 ± 4.9
Lean body mass, arms (kg)	4.68 ± 1.1	4.86 ± 1.6
Lean body mass, legs (kg)	14.88 ± 3.4	14.91 ± 3.5
hs-CRP (mg/l)	1.5 ± 1.7	1.1 ± 1.4
Monocytes (× 10^9^/l)	0.5 ± 0.1	0.5 ± 0.2
Lymphocytes (× 10^9^/l)	1.8 ± 0.4	1.7 ± 0.6

Data are presented as mean ± SD (age, BMI, lean body mass, monocytes, and lymphocytes) or median ± SD (hs-CRP). No significant differences were observed between groups at baseline

The dietary intake was calculated based on two 24-h recall interviews performed prior to, and during the study period (Table 2). As expected, the intake of protein increased in the protein group, while the intake of carbohydrates and fiber were reduced. In the carbohydrate group the intake of protein (E %), saturated fat, and monounsaturated fat were reduced, and the intake of carbohydrates increased.

**Table 2:** Dietary changes during the intervention in the protein group and the carbohydrate group

	Protein group (*n* = 14)			Carbohydrate group (*n* = 17)		
	Mean (SD )	Δ Mean (SD)^1^	*p* value	Mean (SD )	Δ Mean (SD )^1^	*p* value
Energy (MJ)	6.5 (1.9)	0.8 (1.5)	0.11	7.5 (2.7)	0.04 (1.8)	0.002
Protein (E %)	19.4 (4.2)	4.9 (5.3)	0.006	16.8 (3.9)	− 1.6 (4.1)	0.022
Protein (g/kg body weight)	1.1 (0.4)	0.4 (0.3)	0.010	1.0 (0.3)	− 0.1 (0.3)	0.11
Total fat (E %)	35.3 (6.0)	− 1.1 (8.6)	0.65	40.8 (7.7)	− 9.6 (7.9)	0.13
Saturated fat (E %)	13.4 (2.8)	− 0.4 (3.1)	0.65	16.9 (3.6)	− 4.1 (3.5)	0.045
Polyunsaturated fat (E %)	6.4 (2.4)	− 0.04 (3.2)	0.97	6.4 (2.6)	− 1.7 (2.7)	0.16
Monounsaturated fat (E %)	11.7 (1.6)	− 0.3 (3.8)	0.75	13.6 (3.7)	− 2.7 (3.3)	0.046
Carbohydrates (E %)	41.9 (5.2)	− 10.7 (6.6)	< 0.001	38.1 (7.0)	3.3 (5.3)	0.002
Added sugar (E %)	7.0 (3.8)	− 1.0 (3.1)	0.25	6.2 (3.8)	− 1.1 (3.8)	0.078
Fiber (E %)	2.4 (0.9)	− 0.8 (0.7)	0.002	2.0 (0.4)	− 0.3 (0.5)	0.16
Alcohol (E %)	1.7 (5.1)	− 1.8 (5.3)	0.24	3.3 (5.7)	− 3.5 (5.8)	0.082

Data are presented at baseline in the protein and the carbohydrate group as mean ± SD. Changes from baseline to end of the intervention are presented as mean changes (± SD). Within-group effects from baseline to end of the intervention were analyzed using a paired sample *t* test. ^1^Data on one subject is missing

### Gene expression profiling in PBMCs

**Fig. 1:**
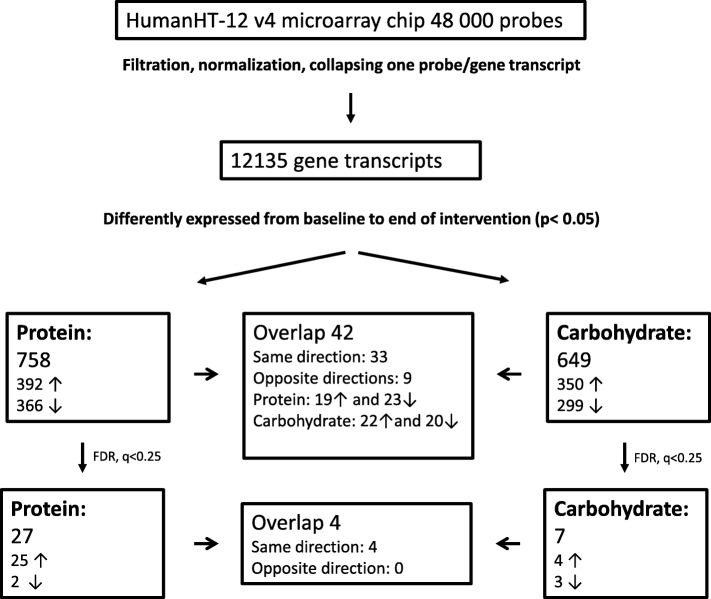
Overview of number of genes regulated after intervention. **I**n total 48,000 probes were on the HumanHT-12 v4 microarray chip. 12,135 were defined as expressed in PBMCs. 758 gene transcripts were changed after protein intake, while 649 gene transcripts were changed after carbohydrate intake (*p* < 0.05). 42 gene transcripts were overlapping in both groups. After adjusting for multiple testing, 27 gene transcripts were significantly changed after protein intake, while seven were significantly changed after carbohydrate intake (FDR, *q*-value < 0.25). Four gene transcripts were overlapping after adjusting for multiple testing

Microarray hybridization was performed on mRNA from PBMCs collected at baseline and after 12 weeks of intervention from all subjects included. From the 48,000 probe sets present at the HumanHT-12 v4 microarray chip, 12,135 unique gene transcripts were expressed in PBMCs in the present study. In total 758 gene transcripts were regulated after intake of protein intake, and 649 gene transcripts were regulated after intake of carbohydrates (*p* < 0.05) (Additional file 1 : Table S1 and Additional file 2: Table S2, respectively), with approximately equal number up- and downregulated gene transcripts (Fig. 1). Only 42 gene transcripts were overlapping between the two groups (Additional file 3: Table S3) (Fig. 1). Among the overlapping genes 19 and 22 gene transcripts were upregulated after intake of protein and carbohydrates, respectively, while 23 and 20 gene transcripts were downregulated in the respective groups (Additional file 3: Table S3). Nine gene transcripts were regulated in opposite direction in the two groups (Fig. 1) (Additional file 3: Table S3).

After adjusting for multiple testing (FDR, *q*-value < 0.25), the expression of 27 gene transcripts were changed from baseline to end of the intervention in the protein group (Table 3), and seven gene transcripts were changed in the carbohydrate group (Table 4).

**Table 3:** Genes significantly regulated after intake of protein (FDR, *q* < 0.25)

Gene symbol	Name	Biological process^a^	Log FC	*p* value	FDR
ZNF683	Zinc Finger Protein 683	Adaptive and innate immune response in NK-cells, transcription	0.367	6.75E-05	0.112
ZNF543	Zinc finger protein 543	Transcription	0.362	1.17E-07	0.001
MICA/B	MHC Class I Polypeptide-Related Sequence A/B	Immune response, T-cell mediated cytotoxicity	0.324	3.68E-05	0.074
KIR2DL1	Killer Cell Immunoglobulin Like Receptor, Two Ig Domains And Long Cytoplasmic Tail 1	Natural killer cell inhibitory signaling pathway	0.321	7.80E-05	0.112
CCL4L2	C-C Motif Chemokine Ligand 4 Like 2	Inflammatory response	0.315	1.01E-04	0.117
KIR2DL4	Killer Cell Immunoglobulin Like Receptor, Two Ig Domains And Long Cytoplasmic Tail 4	Immune response	0.296	3.51E-04	0.228
FGFBP2	Fibroblast Growth Factor Binding Protein 2	Growth factor binding	0.294	2.29E-04	0.214
HOPX	HOP homeobox	Transcription	0.289	2.93E-05	0.073
MMP23B	Matrix Metallopeptidase 23B	Proteolysis and reproduction	0.287	3.71E-04	0.228
ADGRG1	Adhesion G Protein-Coupled Receptor G1	Cell adhesion and migration, cell proliferation, signal transduction	0.273	3.47E-04	0.228
KLRC3	Killer Cell Lectin Like Receptor C3	Cellular defense response	0.270	8.28E-05	0.112
CLIC3	Chloride Intracellular Channel 3	Regulation of ion transmembrane transport	0.265	5.08E-04	0.228
PRSS30P	Protease, Serine, 30 Pseudogene	Pseudogene	0.261	1.10E-04	0.117
NMUR1	Neuromedin U Receptor 1	Calcium-mediated signaling, chloride transport, G-protein coupled receptor signaling pathway	0.258	4.99E-04	0.228
PTGDR	Prostaglandin D2 Receptor	G-protein coupled receptor signaling pathway, cellular and inflammatory responses	0.249	4.48E-04	0.228
KIR3DL3	Killer Cell Immunoglobulin Like Receptor, Three Ig Domains And Long Cytoplasmic Tail 3	Immune response	0.243	4.20E-04	0.228
GNLY	Granulysin	Antimicrobial humoral immune response mediated by antimicrobial peptide	0.237	3.02E-05	0.073
PRL23A	Ribosomal protein L23a	Cell proliferation	0.222	8.73E-06	0.035
KIR-K36	Killer Cell Immunoglobulin Like Receptor – K36	Defense response	0.222	1.16E-04	0.117
CTSW	Cathepsin W	Immune response	0.221	4.71E-04	0.228
KIR2DS5	Killer Cell Immunoglobulin Like Receptor, Two Ig Domains And Short Cytoplasmic Tail 5	Immune response	0.211	4.68E-04	0.228
HERC3	HECT And RLD Domain Containing E3 Ubiquitin Protein Ligase 3	Protein ubiquitination	0.165	4.85E-04	0.228
CCDC65	Coiled-Coil Domain Containing 65	The protein plays a critical role in the assembly of the nexin-dynein regulatory complex.	0.159	4.40E-04	0.228
ABHD17A	Abhydrolase Domain Containing 17A	Protein palmitoylation	0.156	3.06E-04	0.228
TMEM87B	Transmembrane Protein 87B	Involved in endosome-to-trans-Golgi network retrograde transport.	0.139	2.6E-04	0.228
TLR9	Toll Like Receptor 9	Immune response	-0,199	4.51E-04	0.228
KDM5A	Lysine-specific demethylase 5A	Regulation of histone deacetylase activity, transcription	-0,282	4.59E-07	0.003

^a^Biological process as defined by UniProtKB (https://www.uniprot.org/). log FC; log fold change, FDR; false discovery rate

**Table 4:** Genes significantly regulated after intake of carbohydrates (FDR, *q* < 0.25)

Gene symbol	Name	Biological process^a^	Log FC	*p* value	FDR
MICA/B	MHC Class I Polypeptide-Related Sequence A/B	Immune response, T-cell mediated cytotoxicity	0.410	8.5E-10	5.82E-06
ZNF543	Zinc finger protein 543	Transcription	0.301	1.22E-06	0.003
PRL23A	Ribosomal protein L23a	Cell proliferation	0.260	2.04E-07	0.0006
ANO8	Anoctamin 8	Chloride transport	0.198	5.61E-05	0.114
KDM5A	Lysine-specific demethylase 5A	Regulation of histone deacetylase activity, transcription	-0,424	9.58E-10	5.82E-06
HBG2	Hemoglobin Subunit Gamma 2	Blood coagulation	-0,434	7.92E-05	0.137
S1PR5	Sphingosine-1-Phosphate Receptor 5	G-protein coupled receptor signaling pathway	-0,597	2.51E-08	0.0001

^a^Biological process as defined by UniProtKB (https://www.uniprot.org/). log FC; log fold change, FDR; false discovery rate

Among the regulated gene transcripts (FDR, *q*-value < 0.25), four genes were overlapping in the two groups. In both groups, the expression level of *KDM5A* was significantly downregulated, whereas the expression levels of *RPL23A, ZNF543* and *MICA/B* were upregulated (Table 3 and 4). Additional gene transcripts, altered in the protein group, were primarily involved in the immune response, such as KIR2DLs, KLRC3 and CCL4L2 (Table 3).

**Fig. 2:**
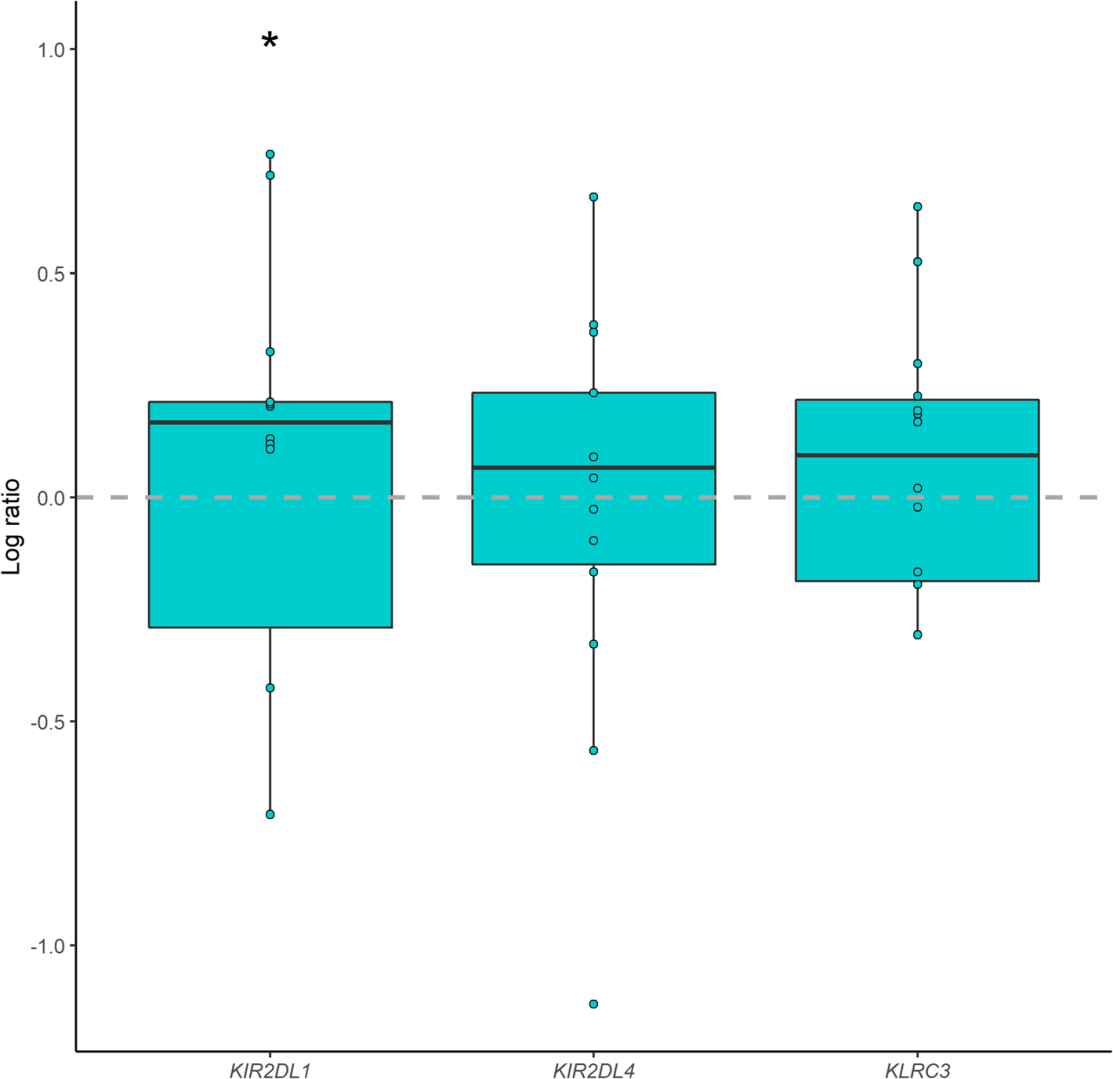
qPCR validation of microarray findings after the 12-week intervention of protein-enriched milk. Change of mRNA expression (log ratio) from baseline to end of study within the protein group. The box represents the 25-75 percentiles (IQR), and the horizontal line shows the median log ratio. The upper and lower whiskers show the largest and smallest values no further than 1.5 x IQR and data beyond the end of the whiskers are outliers and are plotted individually. Differences between the time points were tested with a paired Wilcoxon-Mann-Whitney test. Significantly regulated genes are marked with * (*p* < 0.05)

Four of the most upregulated genes (*KIR2DL1*, *KIR2DL4*, *KLRC3*, and *CCL4L2*) in the protein group (FDR *q*-value < 0.25) (Table 3) were selected for confirmation by RT-qPCR analyses. The mRNA level of *KIR2DL1* (*p* = 0.04) was significantly upregulated after protein intake. The mRNA levels of *KLRC3* and KIR2DL4 were also upregulated after protein intake, but not statistically significantly from baseline (*p =* 0.08, and *p =* 0.67, respectively) (Fig. 2). Although the differences were not significant, the median effect was in the same direction as in the microarray analyses. In contrast to the microarray results, we were not able to detect any expression level of *CCL4L2* with the RT-qPCR method.

No difference in the distribution of monocytes and lymphocytes were observed during the intervention, as previously reported (data not shown) [21].

### Pathway analysis in MetaCore

**Fig. 3:**
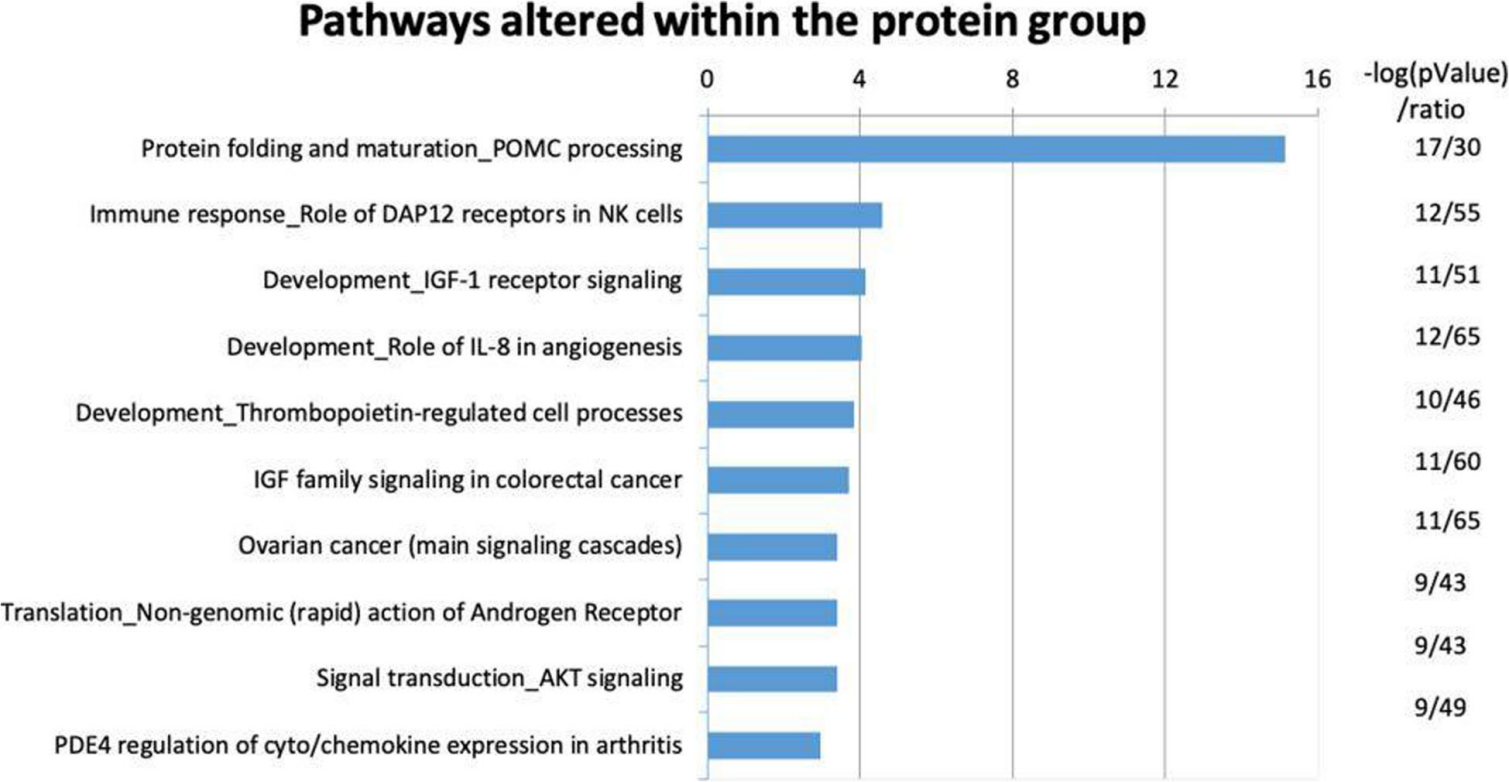
Ten most regulated pathways after the 12-week intervention of protein-enriched milk. -log(pValue) indicates the level of significance, the ratio the number of genes regulated in our sample material compared to the number of genes identified in this specific pathway

To understand further the impact of increased protein intake on metabolic processes, pathway analysis was performed using MetaCore. All differently regulated genes with a nominal *p* value < 0.05 were imported into the software. We identified 96 differently regulated pathways in the protein group (FDR, *q*-value < 0.05) (Additional file 4: Table S4). Pathways related to protein folding and maturation of pro-opiomelanocortin (POMC) processing, immune response in NK cells and development of IGF-1 receptor signaling were among the most ten regulated pathways (Fig. 3). Genes involved in the folding and maturation of POMC processing are among others CAP-Gly Domain Containing Linker Protein (CLIP), joining peptide (JP), the adrenocorticotrophic hormone (ACTH), in addition to several forms of Melanocyte-Stimulating Hormones (MSH) and lipotrophin (LPH). In the present study CLIP1 and POMC were significantly downregulated, while CLIP4 were upregulated, in the protein group (nominal *p* < 0.05) (Additional file 1: Table S1). Pathways were then grouped according to function, e.g. all pathways having “IGF”, “mTORC” and “growth” factor in their names were grouped into one group, and all pathways having “immune” in their names were grouped into the immune response group. Pathways having both “immune” and “apoptosis/survival/signaling transduction” in their names were grouped into the immune response group, others into the apoptosis/survival group and the signal transduction group, respectively. Genes involved in lipid metabolism, and POMC-signaling were grouped into the lipid metabolism, and POMC-signaling groups, respectively (Table 5).

**Table 5:** Regulated pathways (FDR, *q*-value < 0.05) after intake of protein

Pathways	Protein
	Number of regulated pathways (%)
Immune response	35 (36)
IGF1, mTORC1C signaling and growth factors	7 (7)
Cell growth and proliferation	16 (17)
Lipid metabolism	2 (2)
Apoptosis and survival	2 (2)
Signal transduction	18 (19)
POMC-signaling	1 (1)
Others	15 (16)
Regulated pathways (in total)	96

Furthermore, 62 differently regulated pathways were identified in the carbohydrate group (FDR, *q*-value < 0.05). These were related to apoptosis and survival of TNFA-induced caspase-8-signaling, apoptosis and survival of ceramides signaling pathways and IL16 signaling pathways, and are shown in Additional file 5: Table S5 and (flow chart given in Additional file 6: Figure S1).

### IGF-1 in serum and gut peptides in plasma

Based on the results from the pathway analysis in the protein group, we analyzed blood concentrations of IGF-1 and gut peptides. Serum IGF-1 level increased from baseline to the end of the intervention in the protein group and was close to being significant (*p* = 0.08). No change was observed in the level of IGF-1 in the group receiving the isocaloric carbohydrate drink. The change between the groups was also close to being statistically significant (*p* = 0.07) (Table 6). Circulating levels of GIP, GLP-1, PYY, amylin and PP did not differ within or between the groups after 12 weeks (Table 6).

**Table 6:** Effects of protein-enriched milk and isocaloric carbohydrate drink on of IGF-1 and gut peptides

	Protein group (*n* = 14)			Control group (*n* = 17)			*p* value^b^
	Mean (25th–75th )	Mean (25th–75th )	*p* value^a^	Mean (25th–75th )	Mean (25th–75th)	*p* value^a^	
	Baseline	End of study		Baseline	end of Study		
Serum IGF-1 (ng/ml)^c^	70 (57–81)	75 (62–91)	0.08*	74 (60–87)	73 (64–85)	0.68^*^	0.07
Plasma GIP (pg/ml)^d^	136 (79–178)	119 (100–124)	0.51^#^	125 (94–155)	171 (101–200)	0.10^#^	0.10
Plasma GLP-1 (pg/ml)^d^	46 (19–51)	42 (22–45)	0.78^#^	39 (29–47)	43 (31–49)	0.62^#^	0.78
Plasma PP (pg/ml)^d^	287 (150–463)	287 (154–419)	0.55^#^	225 (153–318)	230 (108–244)	0.33^#^	0.32
Plasma Amylin (pg/ml)^d^	397 (313–461)*	393 (213–509)	0.86^#^	372 (213–337)	336 (216–351)	0.92^#^	0.82
Plasma PYY (pg/ml)^d^	347 (297–400)*	351 (285–440)	0.86^#^	340 (285–353)	335 (290–376)	0.94^#^	0.63

^a^Changes within groups were analyzed using ^*^paired sample *t* test and ^#^Wilcoxon signed-ranks test. ^b^Changes between groups were analyzed using ^c^independent sample *t* test and ^d^Wilcoxon-Mann-Whitney test. *n = 13

## Discussion

We investigated genome-wide gene expression changes in PBMCs in older men and women (≥ 70 years) with reduced physical strength and/or performance, before and after a 12-week intervention of increased protein intake. We found that pathways related to protein folding and maturation of POMC processing, immune response in NK-cells and development of IGF-1 receptor signaling were the most regulated pathways after increased protein intake.

POMC is found in many tissues, among them leucocytes [22] and PBMCs [23], being the precursor of several molecules, among them ACTH, b-LPH [22], and α-MSH [23]. POMC-derived peptides are generally known to play an important role in regulating energy homeostasis [24] hunger and satiety [23, 25]. POMC-derived peptides may also exert different effects in different tissues, as post-translational processing events of POMC are common [22, 23]. The functional significance of POMC expression in lymphocytes is unclear, but it is assumed that it forms part of a biochemical loop linking the immune, nervous and endocrine systems [22]. To our knowledge, there has been no studies identifying how diets high in protein may affect the mRNA expression of POMC and POMC related genes in PBMCs. In the present study, CLIP1 and 4, in addition to POMC were significantly regulated (before adjusting for multiple testing), supporting the notion that intake of protein modulated this pathway. High-protein diets have been shown to promote satiety and reduce calorie intake [26] through anorexigenic gut peptides (cholecystokinin, GLP-1, and PYY), but also through hypothalamic pathways involving POMC [27]. Kinzig and colleagues showed that a diet high in protein in rats resulted in significantly increased POMC gene expression, in the hypothalamus [28]. Increased intake of leucine, present in high amounts in dairy products, is an important activator of mTORC1 [29] and has also been shown to increase the mRNA expression of POMC [27].

Among the statistically significantly upregulated genes observed after increased protein intake were Killer Cell Immunoglobulin-like receptors (KIRs) and Killer Cell Lectin Like Receptor C3 (KLRC3) which are closely related to the immune response of TYRO protein tyrosine kinase binding protein (DAP12) receptors in NK cells, as was one of the most regulated pathways within the protein group. These results indicate changes in the activity of NK-cells in the protein group [30].

Seven altered pathways related to IGF and growth hormones signaling after intake of protein were identified. Both IGF and mTORC1 pathways were regulated in PBMCs after the increased protein intake. The regulation of the mTORC1 pathway in PBMC indicated that PBMCs could be used as a model system to study gene expression changes in protein intervention studies, as the most studied pathway regulated by dietary proteins is probably the mTORC pathway [16]. The biological function of the mTORC regulation in PBMCs needs further investigation as this pathway is mainly studied in the metabolic regulation of skeletal muscle. IGF-1 serum levels were increased, but not statistically significant (*p* < 0.08), indicating that increased protein intake might affect metabolism and growth hormone signaling. Previous studies have shown that increased energy intake, intake of essential amino acids [31, 32], and increased intake of milk can increase circulating levels of IGF-1 [33–36]. IGF-1 is an anabolic hormone that has an important function in maintaining skeletal muscle mass across all ages [37], and concentrations of circulating IGF-1 have been shown to decline during aging [31] [38]. The level of IGF-1 and chronic low-grade inflammation may be closely linked [39]. Chronic low-grade inflammation seems to a robust predictor of disability and mortality, even in the absence of clinical disease [40, 41]. Dysregulations of the immune system, including failure in resolving inflammation, may play a role in the etiology and perpetuation of underlying inflammation [42] and sarcopenia [43]. In the present study, we observed that many genes related to the function of NK-cells were upregulated after increased protein intake and several pathways related to immune functions were altered by protein intake.

The major strengths of the present study were the double-blind randomized controlled design with the use of an isocaloric test drink and several data analysis strategies to explore their possible effects. The major limitation is that we cannot rule out that the effect of increased protein intake in the protein group, and the increased carbohydrate intake in the carbohydrate group is solely due to these changes since the subjects had other macronutrient changes during the intervention. Furthermore, we used commercially qPCR with primers that were not identical to the probes used in the microarrays. This may be one reason why we were not able to verify all gene transcripts regulated after protein intake.

## Conclusions

We identified significant changes in gene transcripts and signaling pathways in PBMCs after increased protein intake. Most changes were related to protein folding and maturation of POMC processing, immune response in NK-cells and IGF-1 receptor signaling. Whether these changes in whole genome transcriptome profiles and PBMCs can affect long-term health outcomes by increased protein intake in older adults needs to be further validated.

## Methods

### Subjects and study design

The current study was part of a previously published parallel double-blind, randomized controlled intervention trial, conducted from 2014 to 2015 at Oslo and Akershus University College of Applied Sciences, Norway [9]. Fifty home-dwelling men and women (≥ 70 years) with reduced physical strength and/or performance were found eligible for the study. Invitation letters were sent to 2820 subjects, 438 subjects met to the screening visit of which 388 did not meet the inclusion criteria. In total, 50 subjects were randomized, and 36 subjects concluded the study. Inclusion criteria were either reduced grip strength (< 20 kg in women and < 30 kg in men), gait speed < 1 m/s, time step stair test ≥ 8.4 s or timed five times to sit to stand test > 12.5 s, and willingness to keep the physical activity level stable throughout the study period. Subjects with type I and II diabetes or HbA1 ≥ 6.5%, severe inflammation, chronic obstructive pulmonary disease, high blood pressure (> 180/105 mmHg), acute cardiovascular disease within the last 6 months or a history of cancer the last 3 years were excluded. Subjects with thyroid-stimulating hormone outside reference range (0.2–10 mU/l) were only included if the thyroxine concentration was within the reference value. If a stable dosage of thyroxine treatment, hormone therapy, and antihypertensive drugs had been used prior to inclusion, these drugs were allowed during the study. Subjects were stratified by gender and smoking and allocated within each of the two groups to consume either protein-enriched milk (2 × 0.4 l/day, 2 × 20 g protein/day, 5.0% protein, 4.6% carbohydrates, < 0.1% fat, 167 kJ (39 kcal)/100 g) or an isocaloric carbohydrate drink (2 × 0.4 l/day) for breakfast and evening meal for 12 weeks. The test drinks were identical in color, labeling, and appearance, and were blinded to both the study participants and the study staff. The drinks (0.4 l) were labeled with each participant’s ID-number, and information about day and time to be consumed (morning or evening). The test drinks were picked up by the participants at the study center or delivered to their homes by the study staff. Unopened containers and leftovers were returned to the staff. None of the participants was excluded from the study due to low compliance (≥ 70%). Participants were encouraged to maintain their normal diet and physical activity levels during the study period. The participants registered all physical activity ≥ 30 min during the study period. All subjects completed the study within 12 ± 1 weeks.

### Study products

The protein-enriched drink and the isocaloric carbohydrate drink were produced and provided by TINE SA, Oslo, Norway. The protein-enriched milk is commercial available for sale in Norway, but was not enriched with vitamin D when used in the study. The protein-enriched drink provided on average 167 kJ (39 kcal), 5.0% protein, 4.6% carbohydrates, < 0.1 g fat/100 g. About 80% of the milk protein was casein and the remaining protein was whey protein. The isocaloric, non-nitrogenous control drink was prepared from carbohydrates (sugar, xantan gum and maltosweet®). Calcium was added to the control drink to match the content in the protein-enriched milk, and titan dioxide was added to give the control drink a milky appearance.

Dietary assessments were performed by two 24-h-dietary recalls prior to baseline and two at the end of the intervention [9], reflecting the diet prior to, and during the intervention period. The interviews were performed using an in-house data program (KBS version 7.0) and was linked to the Norwegian food composition table. Dietary supplements were included in the analysis.

Body composition was measured using dual energy X-ray absorptiometry (DXA). The analysis was performed after an overnight fast (≥ 12 h) at the Norwegian School of Sport Sciences, Oslo, Norway, and we used enCORE Software (version 14.10.022, GE Lunar) to estimate lean muscle mass in arms and legs. Muscle mass was defines as the sum of lean body mass of the four limbs [9].

A detailed description of the protocol, participant requirement, and complete list of exclusion criteria, composition of the test drinks, how the 24-h-dietary recalls were performed and compliance are described previously [9].

All subjects provided written informed consent, and the study was conducted according to the Declaration of Helsinki. We received approval for all procedures involving human subjects by the Regional Committees for Medical and Health Research Ethics, Health Region South East, Norway. The study was registered at Clinicaltrials.gov (ID no. NCT02218333).

### Blood sampling and preparation

Blood samples were drawn in the morning after an overnight fast (≥ 12 h) in BD Vacutainer® CPT^TM^ cell preparation tubes with sodium heparin (Becton Deckenson, NJ, USA) at baseline and after 12 weeks. Using CPT^TM^ cell preparation tubes is a well-documented and standardized method to collect mononuclear cells with high purity (above 90 %), and according to the manufacturer, approximately 80% of the cells are lymphocytes and 12% are monocytes. PBMCs were isolated, pellets were stored at − 80 °C before mRNA was extracted using RNeasy Mini Kit (Qiagen) as described elsewhere [21]. RNA quantity was measured using NanoDrop-1000 (NanoDrop Technologies, Inc., Delaware, USA), while RNA quality was checked with Aglient 2100 Bioanalyzer (Agilent Technologies, Inc., California, USA). All sample had a RIN-value above 9, except from one sample that was excluded from further analysis. We also excluded samples from four subjects from further analysis due to high-sensitive C-reactive protein (hs-CRP) > 10 Additional file 7: Figure S2. All RNA samples in the present study are the same as used in our previous study by Gjevestad et al. [21]. In addition, serum samples for the determination of hs-CRP, and EDTA-blood for the differential blood count were collected. The analyses were performed at an accredited laboratory (Fürst Laboratories, Oslo, Norway).

### Microarray hybridization and processing

Following RNA preparation and amplification, using the Illumina Total Prep RNA Amplification Kit (Illumina Inc., California, USA), gene expression measurements were performed by hybridizing the amplified RNA to Illumina HumanHT-12 v4 Expression BeadChip (Illumina Inc., California, USA) according to the manufacturer’s instructions. Samples were scanned using Illumina HiScan System (Illumina Inc., California, USA). The Illumina HumanHT-12 v4 Expression BeadChip provides genome-wide measurements of the expression of over 48,000 probe sets. IlluminaGenome Studio was used to calculate and report a detection *p* value, which represents the confidence that a given transcript is expressed above the background. A gene was defined as expressed when relevant probes with a *p* value below 0.01 were found in more than five samples. After hybridization and scanning, a manual quality control was performed investigating density plots and hierarchical clustering of raw probe densities. One probe per gene (max IQR) was selected for further analysis. The microarray experiments were conducted according to the MIAME (Minimum Information about a Microarray Experiment) guidelines.

### Microarray data analysis

After correcting for background noise, using normexp background correction (neqc filtration, Limma), quantile normalization of the data was performed using the Illumina GenomeStudio software, version 1.7.0. Data were log2-transformed and exported raw (non-normalized) to R (http://www.r-project.org/) for biostatistical analysis using the Linear Models for Microarray Data (Limma) Bioconductor package version 1.1.0. Differential gene expression was estimated by a moderated paired *t* test (Limma) by comparing the relative change from baseline to after the intervention using R software. Gene transcripts that were significantly regulated during the intervention (nominal *p* value < 0.05) were subjected to further gene pathway analysis using MetaCore^TM^ (GeneGo, Thomson Reuters, Michigan, USA). Pathways identified in MetaCore^TM^ with a FDR *q*-value < 0.05 were considered significantly modulated.

### RT-qPCR

RNA was reversely transcribed by a high-capacity cDNA reverse transcription kit (Applied Biosystems). RT-qPCR was performed on an BioRad CFX96 (Bio–Rad Laboratories) with inventoried TaqMan gene expression assays for Killer cell immunoglobulin like receptor, two Ig domains and long cytoplasmic tail 1 (*KIR2DL1,* Hs 04961778_gH), *KIR2DL4* (Hs00427106_m1), Killer cell lectin like receptor C3 (*KLRC3*, Hs01652462_m1), and C-C motif chemokine ligand 4 like 2 (*CCL4L2*, Hs04400556_m1) (Thermo Fisher Scientific). TATA-binding protein (*TBP*, Hs00427620_m1) and glucuronidase beta (*GUSB*, Hs00939627_m1) were chosen as reference genes due to previous experience with these genes in PBMCs [44]. The assays used for the selected genes were chosen due to best coverage according to Thermo Fischer. The relative mRNA level for each transcript was calculated by the ∆∆cycle threshold (Ct) method [45]. Ct values for each target gene were normalized to the average Ct value of the reference genes (Ct_reference_ − Ct_target_ = ∆Ct) and the relative change from baseline to the end of study visits was calculated and expressed as log ratio (∆Ct_end of study_ − ∆Ct_baseline_ = ∆∆Ct).

### Measurements of insulin-like growth factor 1

Serum levels of IGF-1 were measured using an ELISA-kit (R&D Systems Inc., Minneapolis, USA) in accordance with the protocol provided. All samples were measured in duplicates.

### Measurements of gut peptides

Plasma concentrations of glucose-dependent insulinotropic polypeptide (GIP), glucagon-like peptide-1 (GLP-1), pancreatic polypeptide (PP), amylin and peptide YY (PYY) were analyzed using Milliplex Map Kit for human metabolic hormone magnetic bead panel (Cat. no. HMHEMAG-34 K, EMD Millipore Corporation MA, USA). All samples were measured in duplicate along with controls using Bio-Plex 200 system, based on Luminex xMAP technology (Bio-Rad Laboratories Inc., CA, USA) [46].

### Statistical analysis

For normally distributed data, differences between study-groups were performed using the independent samples *t* test, and Wilcoxon-Mann-Whitney test was performed on not normally distributed data. Changes within each study group were performed using the paired sample *t* test or Wilcoxon-Mann-Whitney test. We considered a *p* value of < 0.05 statistically significant when comparing within and between groups. SPSS statistical software, version 25 from Microsoft (SPSS, Inc., USA) and R were used for statistical analyses.

## Supplementary information

**Additional file 1: Table S1.** Genes that were differently expressed (*p*<0.05) after intake of protein. LogFoldChange is calculated by substracting Log2 ratios.

**Additional file 2: Table S2.** Genes that were differently expressed (*p*<0.05) after intake of carbohydrates. LogFoldChange is calculated by substracting Log2 ratios.

**Additional file 3: Table S3.** Overlapping genes regulated in the protein and carbohydrate groups-those in bold were regulated in opposite direction.

**Additional file 4: Table S4.** All pathway maps regulated after intake of protein (FDR, q-value <0.05). The ratio indicates the number of regulated genes in our gene set compared to the total number of genes included in the pathway. Examples of genes included in each pathway are listed in the column to the right. All pathways are manually classified into selected biological processes (immune response, apoptosis and survival, signal transduction and others)

**Additional file 5: Table S5.** All pathways regulated within the carbohydrate group (FDR, *q*<0.05). The ratio indicates the number of regulated genes in our gene set compared to the total number of genes included in the pathway. Examples of genes included in each pathway are listed in the column to the right. All pathways are manually classified into selected biological processes (immune response, apoptosis and survival, signal transduction and others.

**Additional file 6: Figure S1.** Flow chart

**Additional file 7: Figure S2.** Pathways altered within the carbohydrate group

## Data Availability

The datasets generated and analyzed during the current study are available from the corresponding author on reasonable request, pending the authorization to deposit them in a public repository.

## Abbreviations

ACTH: Adrenocorticotrophic hormone
ATF4: Activating transcription factor 4
BMI: Body mass index
CLIP: CAP-Gly Domain Containing Linker Protein
DAP12: TYRO protein tyrosine kinase binding protein
DXA: Dual energy X-ray absorptiometry
FDR: False discover rate
GCN2: General control nonderepressible 2
GIP: Glucose-dependent insulinotropic polypeptide
GLP-1: Glucagon-like peptide-1
hs-CRP: High-sensitive C-reactive protein
IGF-1: Insulin-like growth factor 1
IL: Interleukin
JP: Joining peptide
KDM5A: lysine-specific demethylase 5A
KIRs: killer cell immunoglobulin-like receptors
KLRC3: Killer Cell Lectin Like Receptor C3
LPH: Lipotrophin
MICA/B: MHC Class I Polypeptide-Related Sequence A/B
MSH: Melanocyte-stimulating hormone
mTORC11: Mechanistic target of rapamycin complex 1
NK-cells: Natural killer cells
NKG2D: Natural killer group 2, member D
PBMC: Peripheral blood mononuclear cells
POMC: Pro-opiomelanocortin
PP: Pancreatic polypeptide
PRL23A: Ribosomal protein L23a
PYY: Amylin and peptide YY
TNFA: Tumor necrosis factor alpha
TNFRSF1A: TNF Receptor Superfamily Member 1A
ZNF543: Zinc finger protein 543
