# Supplementary material for: Increased protein intake affects pro-opiomelanocortin (POMC) processing, immune function and IGF signaling in peripheral blood mononuclear cells of home-dwelling old subjects using a genome-wide gene expression approach
Source: Genes Nutr. 2019 Nov 28;14:32. doi: 10.1186/s12263-019-0654-6 (PMC6883584; doi:10.1186/s12263-019-0654-6)
Supplement: Supplementary file 4 — Additional file 4: Table S4. All pathway maps regulated after intake of protein (FDR, q-value <0.05). The ratio indicates the number of regulated genes in our gene set compared to the total number of genes included in the pathway. Examples of genes included in each pathway are listed in the column to the right. All pathways are manually classified into selected biological processes (immune response, apoptosis and survival, signal transduction and others) [file 12263_2019_654_MOESM4_ESM.docx]

Supplementary table 4. All pathway maps regulated after intake of protein (FDR, q-value <0.05). The ratio indicates the number of regulated genes in our gene set compared to the total number of genes included in the pathway. Examples of genes included in each pathway are listed in the column to the right. All pathways are manually classified into selected biological processes (immune response, apoptosis and survival, signal transduction and others).

| **Biological process** | **Pathways maps** | **Ratio** | **FDR** | **Genes in this pathway** |
| --- | --- | --- | --- | --- |
| Other | Protein folding and maturation_POMC processing | 17/30 | 7.037E-16 | POMC (ACTH), POMC (alpha-MSH), POMC (proACTH), POMC, Joining peptide (JP), N-POMC, DA-alphaMSH, beta-MSH, CLIP, POMC (beta-Endorphin extracellular region), gamma-MSH, N-POC, POC (beta-LPH), gamma-LPH, ACTH 1-17, gamma2-MSH, gamma3-MSH |
| Immune response | Immune response_Role of DAP12 receptors in NK cells | 12/55 | 2.697E-05 | KIR2DS2, KIR3DL1, KLRC1, KIR2DL3, KLRD1, PIK3CA, KIR2DL1, KLRC3, KIR2DL4, SOS, HLA-C, VAV2 |
| IGF, mTORC1C signaling | Development_IGF-1 receptor signaling | 11/51 | 7.228E-05 | GSK3A/B, IRS2, I-kB, PIK3CA, ELK1, RPS6KB1, SOS, IBP, NF-kB, FOXO3A, CCND1 |
| Other | Development_Role of IL-8 in angiogenesis | 12/65 | 9.416E-05 | SREBP2 (nuclear), SCAP, FASN, I-kB, SREBP2 precursor, PIK3CA, ELK1, S1PR1, SREBP2 (Golgi membrane), NF-kB, CTSB, IL8RB |
| Other | Development_Thrombopoietin-regulated cell processes | 10/46 | 1.409E-04 | GSK3A/B, CCNDA2, IRS22, CCNA, CCND, PIK3CA, ELK1, RPS6KB1, SOS, AFT1 |
| IGF, mTORC1C signaling | IGF family signaling in colorectal cancer | 11/60 | 2.035E-04 | GSK3A/B, IRS2, CTNNB1, I-kB, PIK3CA, CCND1, RPS6KB1, SOS, DEF6 (IBP), NF-kB, MYB (MYB) |
| Signal transduction | Ovarian cancer (main signaling cascades) | 11/65 | 3.883E-04 | MET (MET), CTNNB1 (CTNNB1), I-kB, PIK3CA (p110-alpha), CCND1, PIK3CA, ELK1, PKA-cat (cAMP-dependent), SOS, NF-kB, ERBB2 ( ERBB2) |
| Other | Translation_Non-genomic (rapid) action of Androgen Receptor | 9/43 | 3.883E-04 | CTNNB1, ADAR, WNT, PIK3CA, RPS6KB1, SOS, NF-kB, ERBB2, FOXO3A |
| Cell growth and proliferation | Signal transduction_AKT signaling | 9/43 | 3.883E-04 | HSP90, GSK3A/B, MET (Met), I-kB, PIK3CA, RPS6KB1, NF-kB, FOXO3A, Cyclin D |
| Immune response | PDE4 regulation of cyto/chemokine expression in arthritis | 9/49 | 1.084E-03 | GSK3A/B, NF-kB p50/p50, CCL4, I-kB, PDE4, PIK3CA, PKA-cat (cAMP-dependent), IFNG, NF-kB1 (p50) |
| Other | Some pathways of EMT in cancer cells | 9/81 | 1.238E-03 | PDGF receptor, JAK1, CTNNB1, I-kB, PIK3CA, AXIN, PDGFD, SOS, PDGFRB |
| Ubiquitination | Proteolysis_Putative SUMO-1 pathway | 7/29 | 1.238E-03 | UBE2E3, RANBP2, NFKBIA, NF-kB, MYB, FASLG (FasR,FAS), SP3 |
| Immune response | Transcription_Sirtuin6 regulation and functions | 10/64 | 1.238E-03 | SREBP2 (nuclear), SCAP, ELOVL6, FASN, SREBP2 precursor, S1PR1, SREBP2 (Golgi membrane), FOXO3A, PRKDC, LKB1 |
| Immune response | Immune response_IFN-alpha/beta signaling via PI3K and NF-kB pathways | 12/94 | 1.400E-03 | JAK1, IRS2, NF-kB2 (p52), I-kB, CCND1, p70 S6 kinases, PIK3CA, NF-kB2 (p100), RSAD2, NF-kB, FOXO3A, NIK(NIK) |
| Signal transduction | Development_Adenosine A3 receptor signaling | 9/53 | 1.400E-03 | CTNNB1, I-kB, Adenosine A3 receptor, CCND1, ELK1, PKA-cat (cAMP-dependent), SOS, NF-kB, GNAQ |
| Immune response | Development_PDGF signaling via STATs and NF-kB | 7/32 | 1.901E-03 | PDGF receptor, JAK1, I-kB, PIK3CA, SOS, NF-kB, PDGFRB |
| Cell growth and proliferation | Development_HGF signaling pathway | 8/47 | 3.408E-03 | MET, CTNNB1, PIK3CA, ELK1, DOCK2, SOS, SDC1, FASR |
| Immune response | Immune response_IL-9 signaling pathway | 7/36 | 3.774E-03 | NF-kB p50/p50, JAK1, IRS2, I-kB, PIK3CA, SOS, NIK |
| Signal transduction | Signal transduction_IP3 signaling | 8/49 | 4.148E-03 | PDGF receptor, MET, Galpha(q)-specific nucleotide-like GPCRs, PIK3CA, ELK1, CaMKK, SOS, GNAQ |
| Signal transduction | Development_ERBB-family signaling | 7/39 | 5.511E-03 | I-kB, PIK3CA, ELK1, SOS, NF-kB, ERBB2, NIK(NIK) |
| Immune response | Apoptosis and survival_APRIL and BAFF signaling | 7/39 | 5.511E-03 | NF-kB p50/p50, NF-kB2 (p52), CCL4, I-kB, NF-kB2 (p100), NIK(NIK), NF-kB1 (p50) |
| Immune response | Immune response_M-CSF-receptor signaling pathway | 10/81 | 5.738E-03 | JAK1, CTNNB1, GAB3, TSAD, CCND1, PIK3CA, ELK1, NF-kB, MAP2K5 (MEK5), SOS1 |
| Immune response | Immune response_IL-11 signaling pathway via MEK/ERK and PI3K/AKT cascades | 9/67 | 6.060E-03 | ADAM10, I-kB, p70 S6 kinases, PIK3CA (p110-alpha), CCND1, NFKBIA, SOS, AFT1, IFNG |
| Immune response | Immune response_T regulatory cell-mediated modulation of effector T cell and NK cell functions | 8/55 | 7.450E-03 | STAT4, CCL4, NKp30, DLL1, Granzyme A, PKA-cat (cAMP-dependent), Perforin, IFNG |
| Other | Colorectal cancer (general schema) | 6/30 | 7.450E-03 | PTCH1, MET, CTNNB1, DLL1, WNT, IL8RB |
| Other | Development_Positive regulation of STK3/4 (Hippo) pathway and negative regulation of YAP/TAZ function | 9/70 | 7.527E-03 | CTNNB1, MARKK, MALS-3, PKA-cat (cAMP-dependent), MPP5, ADRB2,ITCH, FasR, LKB1 |
| Immune response | Immune response_IL-3 signaling via ERK and PI3K | 11/102 | 7.531E-03 | GSK3A/B, PDE4, p70 S6 kinases, PIK3CA (p110-alpha), PIK3CA, IL3RA, ELK1, PKA-cat (cAMP-dependent), SOS, FOXO3A, Slp76 |
| Cell growth and proliferation | Normal and pathological TGF-beta-mediated regulation of cell proliferation | 6/33 | 1.048E-02 | PDGF receptor, CTNNB1, CCND1, AXIN, SOS, PDGFRB |
| Immune response | LRRK2 and immune function in Parkinson's disease | 5/22 | 1.048E-02 | JAK1, I-kB, HSP90 beta, IFNG, NF-kB |
| Immune response | Oxidative stress_Role of Sirtuin1 and PGC1-alpha in activation of antioxidant defense system | 8/60 | 1.048E-02 | TXNRD2, SCPX, NRF2, GSHR, GCL reg, TXN2, FOXO3A, LKB1 |
| Signal transduction | Transcription_Androgen Receptor nuclear signaling | 7/46 | 1.048E-02 | SCAP, CTNNB1, ADAR, WNT, CCND1, PKA-cat (cAMP-dependent), SOS |
| Signal transduction | FGF signaling in pancreatic cancer | 7/46 | 1.048E-02 | CTNNB1, CCND1, PIK3CA, NFKBIA, SOS, NF-kB, SDC1 |
| Cell growth and proliferation | Development_The role of GDNF ligand family/ RET receptor in cell survival, growth and proliferation | 10/92 | 1.067E-02 | CCND12, GDNF, CCND1, PIK3CA, ELK1, NFKBIA, SOS, AFT1, NF-kB, VAV2 |
| Immune response | Development_Leptin signaling via PI3K-dependent pathway | 7/47 | 1.067E-02 | GSK3A/B, IRS2, I-kB, PIK3CA, PKA-cat (cAMP-dependent), CPT1B, LKB1 |
| Signal transduction | Development_PIP3 signaling in cardiac myocytes | 7/47 | 1.067E-02 | GSK3A/B, MET (Met), PIK3CA, RPS6KB1, SOS, FOXO3A, Cyclin D |
| Cell growth and proliferation | Development_PDGF signaling via MAPK cascades | 7/47 | 1.067E-02 | PDGF receptor, PIK3CA, ELK1, PDGF-D, SOS, VAV2, PDGFRB |
| Immune response | Immune response_IL-2 activation and signaling pathway | 7/48 | 1.184E-02 | JAK1, IL2RB, I-kB, PIK3CA, ELK1, SOS, NF-kB |
| Other | Transcription_HIF-1 targets | 10/95 | 1.199E-02 | RORA, ARNT, MXI1, ENG, DEC1 (Stra13), MET (Met), Adipophilin, CX3CR1, MDR1, GPI |
| IGF, mTORC1C signaling | Transport_Macropinocytosis regulation by growth factors | 8/63 | 1.199E-02 | PDGF receptor, EHD4, IRS2, MET, PIK3CA (p110-alpha), SOS, VAV2, PDGFRB |
| Cell growth and proliferation | Cytoskeleton remodeling_Role of PDGFs in cell migration | 5/24 | 1.279E-02 | PDGF receptor, PIK3CA, PDGF-D, VAV2, PDGFRB |
| Other | Main pathways of Schwann cells transformation in neurofibromatosis type 1 | 9/80 | 1.304E-02 | PDGF receptor, CTNNB1, TC21, CCND1, PIK3CA, ELK1, RPS6KB1, ERBB2, PDGFRB |
| Signal transduction | Development_Thromboxane A2 signaling pathway | 7/50 | 1.312E-02 | GSK3A/B, CTNNB1, CCND1, PIK3CA, RAP-1B, RPS6KB1, PKA-cat (cAMP-dependent) |
| Signal transduction | Development_Adenosine A2B receptor signaling | 7/50 | 1.312E-02 | CTNNB1, PIK3CA, ELK1, PKA-cat (cAMP-dependent), SOS, NF-kB, GNAQ |
| Signal transduction | G-protein signaling_H-RAS regulation pathway | 6/37 | 1.385E-02 | MAGI-1(BAIAP1), GDNF, RasGRP4, PIK3CA, SOS, PDGFRB |
| Immune response | Signal transduction_NF-kB activation pathways | 7/51 | 1.416E-02 | NF-kB2 (p52), I-kB, NF-kB2 (p100), NF-kB, NF-kB1 (p105), NIK(NIK), NF-kB1 (p50) |
| Lipid metabolism | Regulation of lipid metabolism via LXR, NF-Y and SREBP | 6/38 | 1.533E-02 | SREBP2 (nuclear), SCAP, FASN, SREBP2 precursor, S1PR1, SREBP2 (Golgi membrane) |
| Other | SLE genetic marker-specific pathways in antigen-presenting cells (APC) | 9/84 | 1.624E-02 | JAK1, IRF8, STAT4, I-kB, S1PR1, IFNG, NF-kB, NIK(NIK), TLR9 |
| Apoptose and survival | Apoptosis and survival_Role of PKR in stress-induced apoptosis | 7/53 | 1.678E-02 | JAK1, I-kB, PP2A regulatory, NFKBIA, IFNG, NF-kB, FasR(FAS) |
| Immune response | Immune response_OX40L/ OX40 signaling pathway | 8/69 | 1.759E-02 | NF-kB2 (p52), I-kB, PIK3CA, NF-kB2 (p100), Perforin, IFNG, NF-kB, NIK |
| IGF, mTORC1C signaling | Main growth factor signaling cascades in multiple myeloma cells | 6/41 | 2.128E-02 | GSK3A/B, IRS2, I-kB, PIK3CA, SOS, NF-kB |
| Lipid metabolism | Regulation of lipid metabolism_Insulin regulation of fatty acid metabolism | 9/89 | 2.263E-02 | IRS2, SCAP, ELOVL6, FASN, PIK3CA, S1PR1, PKA-cat (cAMP-dependent), SOS, ODP2 |
| IGF, mTORC1C signaling | Development_Growth hormone signaling via PI3K/AKT and MAPK cascades | 6/42 | 2.327E-02 | IRS2, Elk-4, PIK3CA (p110-alpha), ELK1, RPS6KB1, SOS |
| Immune response | Signal transduction_Additional pathways of NF-kB activation (in the nucleus) | 5/30 | 2.785E-02 | I-kB, PRMT5, NFKBIA, NIK, NF-kB1 (p50) |
| Other | Development_Ligand-independent activation of ESR1 and ESR2 | 6/44 | 2.805E-02 | PIK3CA (p110-alpha), CCND1, PIK3CA, PKA-cat (cAMP-dependent), SOS, ERBB2 |
| Other | Cell adhesion_Role of CDK5 in cell adhesion | 3/9 | 2.805E-02 | CTNNB1, CDK5, ERBB2 |
| Immune response | Immune response_IL-3 signaling via JAK/STAT, p38, JNK and NF-kB | 9/93 | 2.805E-02 | JAK1, CCND12, ARNT, I-kB, CCND1, PIK3CA, IL3RA, GZMB, NF-kB |
| Immune response | Immune response_TNF-R2 signaling pathways | 6/45 | 3.001E-02 | NF-kB2 (p52), I-kB, PIK3CA, NF-kB2 (p100), NF-kB, NIK(NIK) |
| Cell growth and proliferation | Development_Membrane-bound ESR1: interaction with growth factors signaling | 6/45 | 3.001E-02 | GSK3A/B, CCND1, PIK3CA, ELK1, SOS, ERBB2 |
| Immune response | Immune response_IL-15 signaling | 7/61 | 3.139E-02 | IL2RB, I-kB, PIK3CA, RPS6KB1, SOS, MYB, NIK |
| Other | Regulation of GSK3 beta in bipolar disorder | 6/46 | 3.199E-02 | PP2A regulatory, ADAR, WNT, AXIN, DVL-1, SOS |
| Immune response | Signal transduction_PTMs in IL-17-induced CIKS-independent signaling pathways | 6/46 | 3.199E-02 | JAK1, STAT4, PIK3CA (p110-alpha), PIK3CA, ELK1, NF-kB |
| Signal transduction | Development_Hedgehog signaling | 6/47 | 3.517E-02 | HSP90, PTCH1, CTNNB1, PKA-cat (cAMP-dependent), ITCH, SPOP |
| Cell growth and proliferation | Development_EGFR signaling via small GTPases | 5/33 | 3.622E-02 | PIK3CA, ELK1, SOS, ERBB2, VAV2 |
| Signal transduction | G-protein signaling_G-Protein alpha-q signaling cascades | 5/34 | 3.969E-02 | I-kB, PIK3CA, SOS, NF-kB, GNAQ |
| Other | Development_Melanocyte development and pigmentation | 6/49 | 3.969E-02 | ACTH, alpha-MSH, CTNNB1, PIK3CA, PKA-cat (cAMP-dependent), SOS |
| Immune response | Immune response_Bacterial infections in normal airways | 6/49 | 3.969E-02 | JAK1, I-kB, IFNG, NF-kB, FasR(FAS), NIK(NIK) |
| Signal transduction | Development_PEDF signaling | 6/49 | 3.969E-02 | NF-kB p50/p50, GDNF, PIK3CA, NFKBIA, NF-kB, NF-kB1 (p50) |
| Immune response | Development_G-CSF signaling | 6/49 | 3.969E-02 | JAK1, PIK3CA, SOS, MAP2K5 (MEK5), GFI-1, IL8RB |
| Immune response | Immune response_T regulatory cell-mediated modulation of antigen-presenting cell functions | 7/66 | 4.153E-02 | IRF8, NF-kB2 (p52), NF-kB2 (p100), PKA-cat (cAMP-dependent), IFNG, NF-kB, NIK |
| Cell growth and proliferation | Role of growth factor receptors transactivation by Hyaluronic acid / CD44 signaling in tumor progression | 5/35 | 4.153E-02 | MET, PIK3CA, ERBB2, MDR1, VAV2 |
| Immune response | Development_GM-CSF signaling | 6/50 | 4.153E-02 | I-kB, CCND1, PIK3CA, ELK1, SOS, NF-kB |
| Immune response | PDE4 regulation of cyto/chemokine expression in inflammatory skin diseases | 6/50 | 4.153E-02 | NF-kB p50/p50, PDE4, PKA-cat (cAMP-dependent), NFKBIA, IFNG, NF-kB1 (p50) |
| IGF, mTORC1C signaling | Development_Growth factors in regulation of oligodendrocyte precursor cell proliferation | 7/67 | 4.331E-02 | MET, CCND1, PIK3CA, ELK1, RPS6KB1, SOS, ERBB2 |
| Immune response | Immune response_Regulation of T cell function by CTLA-4 | 5/36 | 4.427E-02 | AP1G1, AP complex 2 medium (mu) chain, PIK3CA, SOS, NF-kB |
| Signal transduction | Development_Hedgehog and PTH signaling pathways in bone and cartilage development | 5/36 | 4.427E-02 | PTCH1, CCND1, CCND1, PKA-cat (cAMP-dependent), GNAQ |
| Immune response | Immune response_IL-12-induced IFNG production | 5/36 | 4.427E-02 | STAT4, I-kB, IFNG, NF-kB, NIK |
| Immune response | Immune response_MIF-mediated glucocorticoid regulation | 4/23 | 4.543E-02 | I-kB, NFKBIA, IFNG, NF-kB |
| Cell growth and proliferation | Development_EGFR signaling via PIP3 | 4/23 | 4.543E-02 | IRS2, PIK3CA, ERBB2, VAV2 |
| Cell growth and proliferation | Development_TGF-beta receptor signaling | 6/52 | 4.543E-02 | SMAD7, ELK1, NFKBIA, SOS, NF-kB, ERBB2 |
| Signal transduction | G-protein signaling_Proinsulin C-peptide signaling | 6/52 | 4.543E-02 | I-kB, CCND1, PIK3CA, ELK1, SOS, NF-kB |
| Other | Mucin expression in CF airways | 7/69 | 4.543E-02 | I-kB, PIK3CA, PKA-cat (cAMP-dependent), NFKBIA, SOS, ERBB2, GNAQ |
| Immune response | Immune response_Gastrin in inflammatory response | 7/69 | 4.543E-02 | I-kB, PIK3CA, ELK1, SOS, MAP2K5 (MEK5), NIK(NIK), GNAQ |
| IGF, mTORC1C signaling | Development_Insulin, IGF-1 and TNF-alpha in brown adipocyte differentiation | 6/53 | 4.655E-02 | IRS2, FASN, RPS6KB1, PKA-cat (cAMP-dependent), ADRB2, INSIG1 |
| Cell growth and proliferation | Development_Endothelin-1/EDNRA signaling | 6/53 | 4.655E-02 | CTNNB1, CCND1, PIK3CA, ELK1, SOS, GNAQ |
| Other | Cell cycle_Influence of Ras and Rho proteins on G1/S Transition | 6/53 | 4.655E-02 | CCND12, MLCP (reg), CCND1, PIK3CA, RPS6KB1, NFKBIA |
| Signal transduction | Development_WNT signaling pathway. Part 2 | 6/53 | 4.655E-02 | NLK, CTNNB1, ADAR, WNT, CCND1, AXIN |
| Signal transduction | Development_Signaling of Beta-adrenergic receptors via Beta-arrestins | 4/24 | 4.684E-02 | GRK5, PKA-cat (cAMP-dependent), SOS, ADRB2 |
| Cell growth and proliferation | CFTR folding and maturation (normal and CF) | 4/24 | 4.684E-02 | ERP29, CANX, HSP90 beta, GANAB |
| Immune response | Immune response_Role of integrins in NK cells cytotoxicity | 5/38 | 4.684E-02 | NKG2A, CD94, SOS, IFNG, ICAM3 |
| Cell growth and proliferation | Transcription_Role of AP-1 in regulation of cellular metabolism | 5/38 | 4.684E-02 | CCND1, GCL reg, IFNG, FASR, HMBS |
| Cell growth and proliferation | Cell cycle_Regulation of G1/S transition (part 1) | 5/38 | 4.684E-02 | PP2A regulatory, CCND1, CCND1, RPS6KB1 |
| Cell growth and proliferation | Development_EGFR signaling pathway | 7/71 | 4.722E-02 | JAK1, I-kB, PIK3CA, ELK1, SOS, NF-kB, ERBB2 |
| Immune response | Immune response_Antigen presentation by MHC class I, classical pathway | 6/54 | 4.722E-02 | HLA-A, CANX, MIC2, GANAB, IFNG, THOP1 |
| Immune response | Immune response_TSLP signalling | 5/39 | 4.969E-02 | JAK1, STAT4, PIK3CA, NFKBIA, NF-kB |
| Signal transduction | Cell adhesion_PLAU signaling | 5/39 | 4.969E-02 | JAK1, MET, PIK3CA, ELK1, SOS |
| Immune response | Immune response_Differentiation and clonal expansion of CD8+ T cells | 5/39 | 4.969E-02 | STAT4, GZMB, PRF1, IFNG, NF-kB |

FDR – false discovery rate
