# Supplementary material for: Increased protein intake affects pro-opiomelanocortin (POMC) processing, immune function and IGF signaling in peripheral blood mononuclear cells of home-dwelling old subjects using a genome-wide gene expression approach
Source: Genes Nutr. 2019 Nov 28;14:32. doi: 10.1186/s12263-019-0654-6 (PMC6883584; doi:10.1186/s12263-019-0654-6)
Supplement: Supplementary file 5 — Additional file 5: Table S5. All pathways regulated within the carbohydrate group (FDR, q<0.05). The ratio indicates the number of regulated genes in our gene set compared to the total number of genes included in the pathway. Examples of genes included in each pathway are listed in the column to the right. All pathways are manually classified into selected biological processes (immune response, apoptosis and survival, signal transduction and others. [file 12263_2019_654_MOESM5_ESM.docx]

Supplementary table 5. All pathways regulated within the carbohydrate group (FDR, q<0.05). The ratio indicates the number of regulated genes in our gene set compared to the total number of genes included in the pathway. Examples of genes included in each pathway are listed in the column to the right. All pathways are manually classified into selected biological processes (immune response, apoptosis and survival, signal transduction and others).

| **Biological processes** | **Pathways** | **Ratio** | **FDR** | **Genes in this pathway** |
| --- | --- | --- | --- | --- |
| Immune response | Apoptosis and survival_TNF-alpha-induced Caspase-8 signaling | 8/43 | 4.645E-03 | PP2A regulatory, PP2A structural, BID, TRADD, PP2A catalytic, CASP3, AKT, Bid |
| Apoptose and survival | Apoptosis and survival_Ceramides signaling pathway | 7/40 | 7.517E-03 | BID, HRAS, TRADD, PP2A catalytic, CASP3, AKT, Bid |
| Immune response | Immune response_IL-16 signaling pathway | 8/55 | 7.517E-03 | IL16, Skp2, PRKCA,CASP3, PRKCA, AKT1 |
| Immune response | Development_Notch Signaling Pathway | 7/43 | 7.517E-03 | NOTCH1, LFNG, CIR, FBXW7, NOTCH1 receptor, NOTCH1 precursor |
| Signal transduction | Development_Prolactin receptor signaling | 8/58 | 7.517E-03 | PRKCD, CREBBP, NMI, HRAS, SOS, SOCS3, AKT, SOCS1 |
| Immune response | Aberrant production of IL-2 and IL-17 in SLE T cells | 8/58 | 7.517E-03 | NOTCH1, PP2A catalytic, CREBP, ETS1, HRAS, SOS, AKT, NOTCH1 precursor |
| Signal transduction | Development_Thyroliberin signaling | 8/60 | 8.254E-03 | PRKCA, PRKCD, CBP, ETS1, GNAQ, HRAS, SOS |
| Immune response | Apoptosis and survival_Caspase cascade | 6/34 | 9.867E-03 | LBR, Bid, TRADD, CASP3, AKT, Bid |
| Signal transduction | Development_CNTF receptor signaling | 6/34 | 9.867E-03 | IL6ST, HRAS, SOS, SOCS3, RHEB2, AKT |
| Signal transduction | Transcription_CREB signaling pathway | 7/49 | 9.867E-03 | LDHA, PRKCA, PRKC (conventional), HRAS, SOS, PP2A catalytic, AKT |
| Other | Role of Tissue factor in cancer independent of coagulation protease signaling | 6/35 | 9.867E-03 | PRKCA, LYN, HRAS, SOS, PAK1, AKT |
| Apoptose and survival | Apoptosis and survival_HTR1A signaling | 7/50 | 9.867E-03 | PP2A regulatory, PP2A structural, HRAS, SOS, PP2A catalytic, CASP3, AKT |
| Other | Development_Gastrin in differentiation of the gastric mucosa | 6/38 | 1.291E-02 | PRKCA, CBP, PRKC (conventional), GNAQ, PKC, GNAQ |
| Cell growth and proliferation | Translation_Regulation of EIF4F activity | 7/54 | 1.291E-02 | IGBP1, HRAS, SOS, PP2A catalytic, RHEB2, PAK1, AKT |
| Other | Development_Differentiation of white adipocytes | 7/54 | 1.291E-02 | CBP, NR1H3, HRAS, SOS, VDR, CD36, INSIG1 |
| Other | Development_Keratinocyte differentiation | 7/56 | 1.503E-02 | NOTCH1, NOTCH2, SMAD2, PRKCA, PRKCD, HRAS, GNAQ |
| Immune response | Immune response_IL-6 signaling pathway via MEK/ERK and PI3K/AKT cascades | 8/74 | 1.503E-02 | PRKD, IL6ST, RPS6, HRAS, SRF, SOS, RHEB2, AKT |
| Immune response | Apoptosis and survival_Apoptotic TNF-family pathways | 6/42 | 1.733E-02 | TNFRSF25, TNFRSF1B, Bid, TRADD, CASP3, Bid |
| Other | Oxidative stress_Activation of NADPH oxidase | 7/59 | 1.774E-02 | PRKCA, PRKCD, PRKC (conventional), PAK1, NCF2, AKT |
| Apoptose and survival | Apoptosis and survival_Anti-apoptotic action of Gastrin | 6/43 | 1.777E-02 | PRKCA, GNAQ, SRF, PAK1, AKT |
| Signal transduction | Cell adhesion_Ephrin signaling | 6/45 | 1.979E-02 | EPHA8, EPHBs, EPHAs, HRAS, EPHB1, PAK1 |
| Other | Development_EPO-induced MAPK pathway | 6/45 | 1.979E-02 | PRKCA, LYN, HRAS, SOS, PAK1, SOS2 |
| Cell growth and proliferation | Development_Gastrin in cell growth and proliferation | 7/62 | 1.979E-02 | PRKCA, PRKCD, GNAQ, HRAS, SOS, PAK1 |
| Cell growth and proliferation | Development_Endothelin-1/EDNRA transactivation of EGFR | 6/46 | 1.979E-02 | PRKCD, HRAS, SOS, RHEB2, AKT, GNAQ |
| Other | Regulation of GSK3 beta in bipolar disorder | 6/46 | 1.979E-02 | PP2A regulatory, Axin, HRAS, SOS, PP2A catalytic, AKT |
| Cell growth and proliferation | Signal transduction_PTEN pathway | 6/46 | 1.979E-02 | TCF, HRAS, SOS, CASP3, RHEB, AKT |
| Immune response | Immune response_MIF - the neuroendocrine-macrophage connector | 6/47 | 2.141E-02 | MIF, CBP, PRKC (conventional), ABCA1, PP2A catalytic, PRKC |
| Signal transduction | Cytoskeleton remodeling_Reverse signaling by Ephrin-B | 5/32 | 2.143E-02 | EPHBs, Axin, HRAS, SOS, PAK1 |
| Cell growth and proliferation | Signal transduction_ERK1/2 signaling pathway | 5/32 | 2.143E-02 | PRKCA, PRKCD, HRAS, SOS, SOS2 |
| Immune response | Apoptosis and survival_Granzyme B signaling | 5/33 | 2.359E-02 | NOTCH1, tBid, CASP3, LMNB1, Bid |
| Immune response | Immune response_IL-11 signaling pathway via MEK/ERK and PI3K/AKT cascades | 7/67 | 2.359E-02 | SFK, IL6ST, RPS6, HRAS, SOS, CASP3, AKT |
| Other | Development_Thromboxane A2 signaling pathway | 6/50 | 2.429E-02 | TCF, PRKC (conventional), GNAQ, HRAS, PKC, AKT |
| Other | Rheumatoid arthritis (general schema) | 6/50 | 2.429E-02 | MHC class II beta chain, HLA-DRB, TNF-R2, HLA-DRB1, HLA-DRB4, CD86 |
| Apoptose and survival | Apoptosis and survival_Role of CDK5 in neuronal death and survival | 5/34 | 2.429E-02 | PRKCD, HRAS, SOS, CASP3, AKT |
| Immune response | Immune response_Gastrin in inflammatory response | 7/69 | 2.488E-02 | PRKCA, PRKCD, GNAQ, HRAS, SOS, AKT |
| Other | Some pathways of EMT in cancer cells | 6/51 | 2.488E-02 | Axin, HRAS, LEF1, TRADD, SOS, AKT |
| Immune response | Development_TGF-beta-dependent induction of EMT via SMADs | 5/35 | 2.555E-02 | NOTCH1, E2A, SMAD2, ETS1, LEF1 |
| Other | Development_EPO-induced Jak-STAT pathway | 5/36 | 2.691E-02 | LYN, HRAS, SOS, SOCS3, SOCS1 |
| Signal transduction | HBV signaling via protein kinases leading to HCC | 5/36 | 2.691E-02 | PRKCA, PRKC (conventional), HRAS, SOS, PRKC |
| Immune response | Immune response_Regulation of T cell function by CTLA-4 | 5/36 | 2.691E-02 | LYN, CD86, SOS, PP2A catalytic, AKT |
| Signal transduction | Aberrant B-Raf signaling in melanoma progression | 6/55 | 3.249E-02 | NOTCH1, CREBBP, AURKB, AKT3, RHEB2, AKT(PKB) |
| Other | Proteolysis_Putative ubiquitin pathway | 4/23 | 3.284E-02 | UEV1A, CHIP, ATXN3, FBXW7 |
| Other | Cytoskeleton remodeling_Regulation of actin cytoskeleton organization by the kinase effectors of Rho GTPases | 6/58 | 4.048E-02 | WRCH-1, PAK, SPTB, PAK1, CDC42, PRK1 |
| Immune response | Development_Leptin signaling via JAK/STAT and MAPK cascades | 4/25 | 4.048E-02 | LEPR, HRAS, SOS, SOCS3 |
| Cell growth and proliferation | Apoptosis and survival_Apoptotic Activin A signaling | 4/25 | 4.048E-02 | ALK-4, SMAD2, HRAS, AKT |
| Lipid metabolism | Regulation of lipid metabolism_G-alpha(q) regulation of lipid metabolism | 6/59 | 4.048E-02 | ABCG1, NR1H3, GNAQ, ABCA1, PRKC |
| Immune response | Immune response_Neurotensin-induced activation of IL-8 in colonocytes | 5/41 | 4.048E-02 | PRKCA, GNAQ, HRAS, SOS, PAK1 |
| Immune response | Signal transduction_PTMs (phosphorylation and acetylation) in TNF-alpha-induced NF-kB signaling | 5/41 | 4.048E-02 | PRKCA, PRKCD, CBP, TRADD, DGKA |
| Cell growth and proliferation | Translation_Insulin regulation of translation | 5/42 | 4.276E-02 | RPS6, HRAS, SOS, RHEB2, AKT |
| Immune response | Development_NOTCH1-mediated pathway for NF-KB activity modulation | 4/26 | 4.276E-02 | NOTCH1, CIR, NOTCH1 receptor |
| Other | Neurophysiological process_Dopamine D2 receptor transactivation of PDGFR in CNS | 4/26 | 4.276E-02 | PP2A regulatory, PP2A structural, PP2A catalytic, PRKC |
| Other | Hypoxia-induced EMT in cancer and fibrosis | 3/13 | 4.276E-02 | NOTCH1, E2A, ETS1 |
| Signal transduction | Development_c-Kit ligand signaling pathway during hemopoiesis | 6/61 | 4.276E-02 | PRKCA, LYN, HRAS, SOS, AKT, SOCS1 |
| Immune response | Immune response_IL-7 signaling in B lymphocytes | 5/43 | 4.288E-02 | E2A, LYN, IL7RA, HRAS, AKT |
| Other | Development_EPO-induced PI3K/AKT pathway and Ca(2+) influx | 5/43 | 4.288E-02 | HBG1, HBB, LYN, HBA1, AKT |
| Immune response | Apoptosis and survival_TNFR1 signaling pathway | 5/43 | 4.288E-02 | TRADD, CASP3, Bid |
| Signal transduction | Muscle contraction_Oxytocin signaling in uterus and mammary gland | 6/62 | 4.316E-02 | PRKCA, PRKC (conventional), HRAS, PP2A catalytic, PRKC, GNAQ |
| Immune response | Apoptosis and survival_Anti-apoptotic TNFs/NF-kB/IAP pathway | 4/27 | 4.374E-02 | TNFRSF12, TNFRSF8, TNFRSF1B, TRADD |
| Signal transduction | Development_Angiotensin signaling via PYK2 | 5/44 | 4.443E-02 | PRKCD, HRAS, SOS, PAK1, GNAQ |
| Cell growth and proliferation | Transport_Macropinocytosis regulation by growth factors | 6/63 | 4.443E-02 | EHD4, LEPR, HRAS, SOS, PAK1, AKT |
| Other | Development_Role of CNTF and LIF in regulation of oligodendrocyte development | 4/28 | 4.730E-02 | IL6ST, SOCS3, CASP3, AKT |
| Signal transduction | Development_Activation of ERK by Alpha-1 adrenergic receptors | 5/45 | 4.730E-02 | TGM2, PRKCA, PRKCD, GNAQ, HRAS |

FDR – false discovery rate
