# Supplementary figures and images for: Increased protein intake affects pro-opiomelanocortin (POMC) processing, immune function and IGF signaling in peripheral blood mononuclear cells of home-dwelling old subjects using a genome-wide gene expression approach
Source: Genes Nutr. 2019 Nov 28;14:32. doi: 10.1186/s12263-019-0654-6 (PMC6883584; doi:10.1186/s12263-019-0654-6)

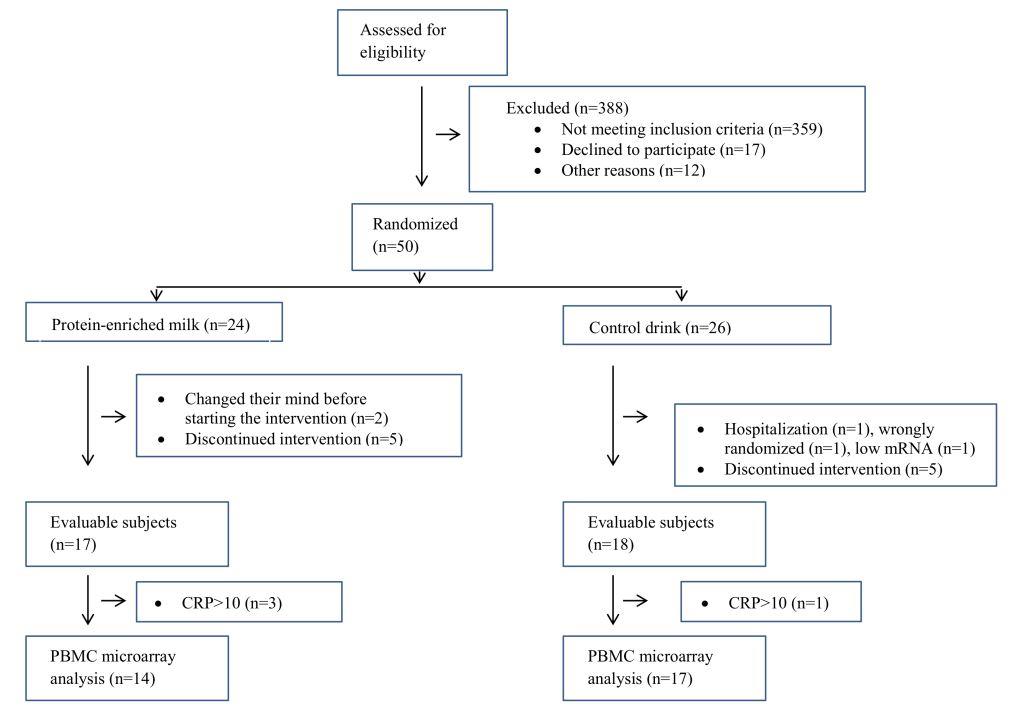

Supplement: Supplementary file 6 — Additional file 6: Figure S1. Flow chart [file 12263_2019_654_MOESM6_ESM.jpg]

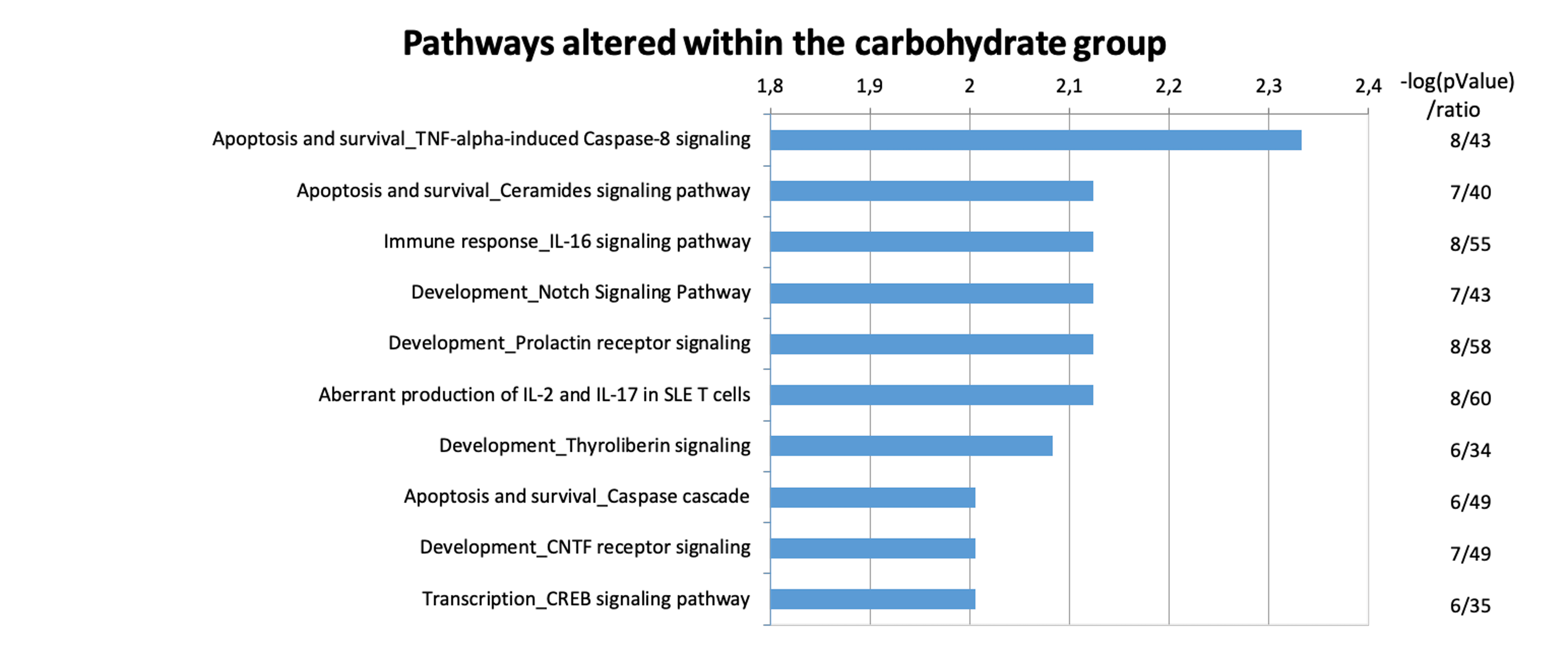

Supplement: Supplementary file 7 — Additional file 7: Figure S2. Pathways altered within the carbohydrate group [file 12263_2019_654_MOESM7_ESM.tiff]
